# The experiences and needs of Australian medical oncologists in integrating comprehensive genomic profiling into clinical care: a nation-wide survey

**DOI:** 10.18632/oncotarget.28076

**Published:** 2021-10-12

**Authors:** Subotheni Thavaneswaran, Mandy Ballinger, Phyllis Butow, Bettina Meiser, David Goldstein, Frank Lin, Christine Napier, David Thomas, Megan Best

**Affiliations:** ^1^Garvan Institute of Medical Research, Darlinghurst, NSW 2010, Australia; ^2^The Kinghorn Cancer Centre, St Vincent’s Hospital, Darlinghurst, NSW 2010, Australia; ^3^St Vincent’s Clinical School, University of NSW, Darlinghurst, NSW 2010, Australia; ^4^School of Psychology, Psycho-Oncology Co-Operative Research Group (PoCoG), The University of Sydney, Sydney, NSW 2006, Australia; ^5^Prince of Wales Clinical School, University of NSW, Sydney, NSW 2052, Australia; ^6^Institute for Ethics and Society, University of Notre Dame Australia, Sydney, NSW 2007, Australia

**Keywords:** comprehensive genomic profiling, precision medicine, targeted treatment selection using genomics, communication, clinicians' experiences with utilising genomic findings in a pan-cancer setting to inform treatment selection

## Abstract

Purpose: Comprehensive genomic profiling (CGP) is increasingly used to guide cancer therapy. This study aimed to characterise oncologists’ experiences and needs when utilising genomic results.

Materials and Methods: An electronic survey distributed nation-wide to practising medical oncologists in Australia explored oncologists’ experiences with consenting, interpreting and communicating CGP results to patients.

Results: The survey was completed by 108 of 333 oncologists (32%) and most respondents (*n* = 97, 90%) had referred patients for CGP. Using a 100-point visual analogue scale score [VAS], where higher values indicate greater confidence, most oncologists were confident consenting patients for referral [median 75 (Interquartile range, IQR: 53–85), discussing CGP results (median VAS: 70, IQR: 51–80), but significantly less confident discussing secondary germline findings (median VAS: 56, IQR 30–70, *p* < 0.001). Confidence with pursuing therapies based on CGP results increased with clinical experience (mean VAS increases by 4.8 per 5 years of experience, *p* < 0.001). Most oncologists (*N* = 68, 63%) reported wanting assistance with interpretation of CGP and patient-centric resources to aid communication with patients.

Conclusions: Oncologists are integrating genomics into clinical care, but only display moderate confidence in communication and changing management accordingly. The development of patient- and clinician- targeted resources may assist with routine utilisation of CGP results in cancer care.

## INTRODUCTION

Medical oncologists were among the first clinicians to integrate genomics into clinical care. Traditionally, this involved single gene testing or small gene panels in select cancer histotypes with established predictive, prognostic or therapeutic implications [1, 2]. The implementation of comprehensive genomic profiling (CGP) has however been much more complex, with most gene panels comprised of hundreds of genes and applied to populations unselected by cancer type [3]. The rapidity of the evolution of CGP places great responsibility on oncologists to discuss implications of testing, requiring familiarity with ever-expanding genomic and therapeutic knowledgebases [4, 5]. CGP can also reveal secondary germline findings [6–8], with up to 18% of patients having germline variants in cancer susceptibility genes identified [9]. The interpretation and management of germline findings outside a classic familial cancer context creates new challenges for oncologists, patients and their families [10, 11].

Understanding oncologists’ perspectives on their role and needs when communicating results to patients is important to the successful integration of CGP into routine care. Few studies have explored clinicians’ experiences utilising CGP [5, 12–14]. These data demonstrate varied uptake of genomic sequencing, and a lack of confidence amongst oncologists incorporating genomic results into clinical care [3, 15–20]. The uptake of CGP was greatest in high-patient volume and academic centres [15, 20] and amongst clinicians with training in genomics [3]. In an Australian context [3, 15], the Australian Pancreatic Genome Initiative published a practical framework for communicating genomic research results [20], including informed consent, analytical validity, clinical relevance of results and results communication [20]. How this guidance has impacted clinical practice is not clear.

Here we aimed to understand Australian oncologists’ views and experiences in integrating CGP into clinical care, assessing current practice and confidence in performing CGP-related tasks, and potential resource needs.

## RESULTS

### Survey participants

Between July 2018 and January 2019, a total of 108 oncologists completed the survey (participation rate = 32%) (Table 1). Males predominated (*n* = 62, 57%, *p* = 0.15, one-sample proportion test), with most participants having less than 15 years of experience (*n* = 76, 70%), most were aged under 50 years (*n* = 83, 77%) and worked within urban-based practices (*n* = 90, 83%) in New South Wales (*n* = 68, 63%).

**Table 1:** Participating oncologists’ demographics

Characteristic	Frequency	Percentage (%)
**Gender**		
Male	62	57.4
Female	46	42.6
**Age**		
20–29 years	1	0.9
30–39 years	49	45.4
40–49 years	33	30.6
50–59 years	11	10.2
60–69 years	14	13.0
**Urban or regional**		
Urban	90	83.3
Regional	18	16.7
**Practicing State**		
Australian Capital Territory	5	4.6
New South Wales	68	63.0
Northern Territory	2	1.9
Queensland	7	6.5
South Australia	9	8.3
Tasmania	1	0.9
Victoria	11	10.2
Western Australia	5	4.6
**Years worked as an oncologist**		
0–4 years	32	29.6
5–9 years	27	25.0
10–14 years	17	15.7
15–19 years	7	6.5
20–24 years	10	9.3
25–29 years	5	4.6
30–34 years	8	7.4
35–39 years	2	1.9
>40 years	0	0

### Experience with genomic testing

Most oncologists (*n* = 97, 89.6%) had referred patients for CGP, and most had experience referring patients to a Family Cancer Centre (FCC) based on family history (*n* = 103, 95.4%, Table 2). No significant associations were found between participant characteristics (gender, age, practicing state, or clinical experience) and experience with CGP (multiple regression, *p* = 0.58, Supplementary Table 1). Approximately two-thirds of oncologists (*n* = 70, 64.8%) also had experience with patients undergoing direct-to-consumer (DTC) tumor testing (mode: 1–5 patients) through a commercially available sequencing panel. DTC refers to CGP that is instigated and coordinated by the patient and involves them working directly with the service provider.

**Table 2:** Experience with testing

Experience referring patients for molecular tumor profiling	Number of patient referrals made	Number (%) of participants
	Never	11 (10.2)
	1–5	29 (26.9)
	6–10	33 (30.6)
	11–20	14 (13.0)
	>20	21 (19.4)
Experience with direct to consumer (DTC) *tumor* testing	Number of patients having had DTC *tumor* testing	Number (%) of participants
	Never	40 (37.0)
	1–5	47 (43.5)
	6–10	14 (13.0)
	11–20	5 (4.6)
	>20	2 (1.9)
Experience with DTC *germline* testing	Any experience with DTC *germline* testing?	Number (%) of participants
	No	96 (88.9)
	Yes	12 (11.1)
Experience with referring patients to a Family Cancer Centre	Number of patient referrals made	Number (%) of participants
	Never	5 (4.6)
	1–5	10 (9.3)
	6–10	12 (11.1)
	11–20	18 (16.7)
	>20	63 (58.3)

### Role of oncologist in ensuring clinical suitability to order tumor profiling

Nearly all oncologists felt it was their responsibility to ensure patients’ clinical suitability to undergo CGP (*n* = 105, 97%). Most respondents indicated that adequate life expectancy (≥ 12 weeks, *n* = 78, 74%), organ function (*n* = 99, 94%), and reasonable Eastern Cooperative Oncology Group (ECOG) performance status (*n* = 89, 95%) were prerequisites to referring patients. Twenty-three oncologists (21%) indicated they would initiate CGP only to access therapy with reasonable efficacy in the relevant context, in the absence of standard treatment options or where significant comorbidities precluded standard treatment, and where the patient had a full understanding of the potential financial implications.

### Obtaining informed consent

Most oncologists (82.4%) believed they had a role in obtaining patient consent for CGP (median VAS 75, IQR: 53–85), but were less comfortable obtaining consent for germline testing (median VAS 44). Oncologists were confident in discussing the process of CGP (median VAS: 75, IQR: 50–86), identifying a ‘therapeutically actionable finding’ (median VAS: 70, IQR: 53–80), and communicating genomic profiling results (median VAS: 70, IQR: 51–80). They were significantly less confident in discussing germline findings (median pairwise difference: –10, IQR: –30–0, *p* < 0.001, Wilcoxon test with continuity correction).

### Pursuit of therapy using CGP results

Most oncologists (60%) would advise against pursuing an unproven targeted therapy based on CGP prior to standard of care treatment, but most (91%) would consider this option in the treatment-refractory setting. Most oncologists (84.3%) still required some evidence to pursue matched therapy outside a trial setting. Relevant evidence included clinical effectiveness in any cancer type (18.7%), where the efficacy was linked to a biomarker (25.3%), especially in the relevant tumor type (56%). Eleven (12.1%) participants reported that case reports/series constituted sufficient evidence. Interestingly, oncologists’ confidence to pursue an unproven targeted therapy increased with years of clinical experience, with every 5 years of clinical experience associated with a mean 4.8 points (95% CI: 2.0–7.5) increase in confidence to pursue an unproven therapy (*p* = 0.001, Supplementary Table 2). Additional patient, clinician and treatment factors were the absence of standard treatment options (*n* = 2, 2.2%), the rarity of the cancer and biomarker, and lastly patient preferences (*n* = 1, 1.1%) (Table 3).

**Table 3:** Key factors in the use of genomic results to access targeted therapy

Patient factors	Clinician factors	Treatment factors
Patient wellness/ECOG	Training/education/experience	Cost
Patient preference/interest	Acceptable level of evidence for clinical benefit	Availability/access program/clinical trial
Age	Therapeutic Goods Administration approval	Safety/toxicity
Financial status	Hospital policy	
Rare cancer histology		
Lack of other available therapies		
Informed patient consent		

### CGP report

Ninety-eight of 108 participants (90.7%) had experience with CGP reports, and felt it was their role to communicate these results to patients. This includes communication of CGP results initiated by patients (DTC). Most oncologists (*n* = 103, 95.4%) wanted reports to include genes on the assay and a statement of therapy recommendations (*n* = 93, 86.1%), and most (80/98, 82%) felt there was sufficient information to understand the results. Areas for suggested improvement included greater explanation and evidence-based ranking of gene prioritisation, clinical significance and treatment recommendations. Some clinicians did not want recommendations to an inaccessible treatment, including international clinical trials, or detailed explanation of the assay. A significant fraction (49, 46%) wanted only those variants that are clinically actionable, while 39 (36.1%) wanted all genetic variants, and 20 (18.5%) only those variants that are therapeutically actionable. Importantly, most participants (*n* = 99, 91.7%) would value a list of matched trials for Australian patients. Acceptable sources of supportive evidence for treatment recommendations included a molecular tumor board (MTB), published evidence, and genomic database references. Nearly all oncologists (*n* = 104, 96.3%) wanted the report to include referral recommendations if potential germline variants in cancer predisposition genes were indicated.

### Assistance with interpreting CGP results

Most oncologists (*n* = 68, 62.9%) reported they needed, or wanted assistance with interpreting CGP results. Currently used sources of support included an oncology colleague (80, 74.1%), the local FCC (50, 46.3%), while 33 (30.6%) reported ‘other’, including reporting institution, own research, molecular pathologist or local expert. Most oncologists (*n* = 77, 71.3%) were significantly less confident in discussing inherited cancer risk; median VAS was 49.5 (IQR: 30.0–63.5). As indicated by very few responses (*N* = 8, 7.4%) with VAS in the top quintile (≥ 80), only a minority of oncologists were confident in discussing secondary germline findings with patients and their family following tumor profiling.

### Resources to assist with communicating results to patients

**Figure 1:**
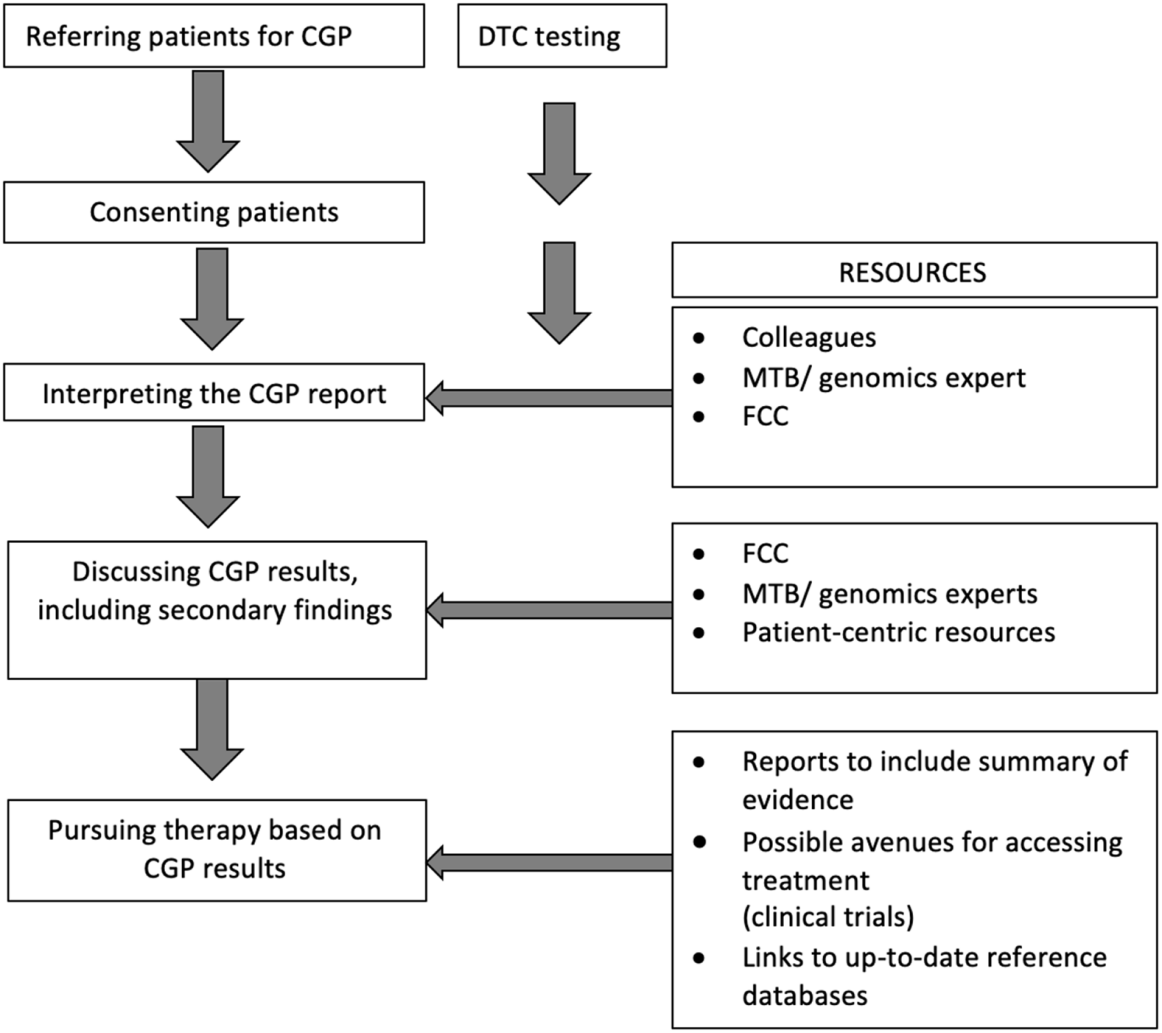
Steps followed by oncologists in the clinical integration of genomic information and resources used or sought. Abbreviations: CGP: comprehensive genomic profiling, DTC: direct to consumer; FCC: Family Cancer Centre; MTB: molecular tumor board.

Participants were asked to identify potential sources of support in communicating the results of CGP to patients and their families (Table 4). The highest priority resources identified included patient information sheets, decision aids, an FCC helpline for patients and clinicians, and a text-based primer of genomics. More experienced oncologists indicated a trend towards lower preference for clinician-based resources (*P* = 0.04, Supplementary Table 3). *p* = 0.016). Figure 1 provides a synoptic overview of steps followed by oncologists in the clinical integration of CGP results, synthesizing survey responses.

**Table 4:** Preferred resources

Resource	Visual analogue scale Median (IQR)
FCC helpline for patients	69 (50–80)
Downloadable patient information sheets on genomic testing and its implications	80 (70–95)
Downloadable patient decision aids	78 (68–90)
FCC helpline for clinicians	70 (50–80)
An online primer on genomics (text)	65 (50–79)
An online primer on genomics (video)	48 (21–69)
**Additional suggested resources**	
Evidence – reference to published literature on genetic variants’ clinical relevance	
An app or database of current evidence for therapeutic efficacy per biomarker	
Update on variant calling	
Case study seminars by an experienced clinician	
Structured reports including types of variants and suggested clinical advice	
Compendium of relevant clinical and basic science information for clinician	

## DISCUSSION

Oncologists clearly identify a role in consenting patients for CGP, and feel comfortable with the process, and the probability of identifying ‘therapeutically actionable findings’ and results. Even when testing was instigated by patients, most oncologists felt it was their role to discuss results, particularly when there were treatment implications. Earlier studies have similarly shown greater use of genomic sequencing in large volume, and academic centres [19]. While our survey did not directly question genomic knowledge, it addressed other components of the genomic confidence scales applied in earlier studies [3, 12], including the ability to discuss CGP results with patients and apply them to treatment recommendations. A study conducted through a single, tertiary, comprehensive cancer care center reported 90% of physicians feeling somewhat/ very confident with their ability to explain genomic concepts to patients and ~75% feeling somewhat/ very confidence with their ability to make treatment recommendations based on genomic information [12]. Comparable levels of variation in ‘genomic confidence’ were seen in our study, with 91% of oncologists believing it was their role to communicate CGP results to patients, but 63% wanting assistance with translating genomic information into treatment recommendations. Our study highlighted the need for greater support for oncologists in interpreting and communicating CGP results effectively. The predominant reason for oncologists rejecting the role to discuss CGP results related to either a lack of expertise to interpret, or uncertainty about the clinical relevance of the finding. Specifically, most participants would like greater guidance as to test validity and the strength of therapeutic recommendations. Clinicians particularly lack confidence in extrapolating treatment decisions based on genomic profiling results outside cancer types in which the evidence for efficacy has been established [21].

Providing a transparent, evidence-based therapy recommendation is key to effectively translating CGP results to the bedside. The confidence of oncologists in applying CGP results to initiate off-label therapies increases with clinical experience, but the majority would pursue investigational agents only in a clinical trial setting. Most oncologists would advise against unproven therapies before standard treatment options had been exhausted. With the rapid evolution of CGP and biomarker-directed therapies, practical guidelines appear to be needed to assist with consistent and effective therapeutic decision-making. Oncologists feel less comfortable dealing with incidental germline findings, despite the high frequency of such results in the context of tumor profiling. This is in keeping with previous studies [22]. Most oncologists confined their role to discussing the potential for secondary findings during consent to CGP. With considerable variation, most participants felt the level of detail on the genomic report was sufficient for interpretation. Many felt the report contained too much detail, wanting only those cancer gene variants with actionability. A list of genes tested during CGP, a statement relating gene variants to recommended therapies, available trials in an Australian context, and a recommendation for referral to the FCC were all valued components of the report. Oncologists expressed a higher preference for patient-centred resources to assist with communicating test results.

Our study has certain limitations. Firstly, this study was performed in the setting of a major national precision oncology trial, the Molecular Screening and Therapeutics (MoST) Program [16]. Despite the breadth of the survey, it is likely to have over-sampled oncologists having referred patients to the Molecular Screening and Therapeutics (MoST) Program for CGP, and therefore not be representative of Australian medical oncologists as a whole. Thus, our findings may over-estimate oncologists’ experience with CGP. If so, the need for support and resources for implementation of CGP in clinical practice is likely even greater. Secondly, in a rapidly evolving field, these findings may change substantially if repeated in 2021. Ongoing surveillance of the oncology landscape is warranted to determine the relevance to clinical experience today.

In conclusion, this study provides insight into how Australian oncologists are coping in the era of precision medicine, and their perceptions of potential barriers to the clinical translation of genomic output. Medical oncologists appear to have accepted CGP as an element of clinical care delivery, and feel responsible for discussing the interpretation and management implications with their patients. Understanding their needs is the foundation for establishing appropriate and effective supports and resources. These considerations will be increasingly important with the rapid evolution and integration of genomic-guided clinical management of cancer patients.

## MATERIALS AND METHODS

### Study type

The study employed a cross-sectional survey design. Ethics approval was obtained from St Vincent’s Hospital Human Research Ethics Committee, file number 18/083.

### Survey design

The online survey was developed based on discussions within an expert advisory group comprising genomics researchers, molecular pathologists, medical oncologists and psycho-oncologists. The survey comprised 25 items eliciting views/experiences/needs related to utilising CGP results and participant demographic data. A 100-point visual analogue scale score (VAS) was used to assess perceived role and level of confidence in utilising CGP results, with higher values indicating greater confidence. Open-ended questions elicited more detail regarding oncologists’ rationale for views and needs. The survey listed a range of options for participants to indicate their preferred resources to assist with communicating results to patients. The survey was piloted with ten oncologists, and minimal changes were made (Supplementary Data).

### Sampling and recruitment

The Medical Oncology Group of Australia membership (*n* = 333) was sent a single email invitation and weblink in July 2018. Study investigators were blinded to the identity and responses of survey recipients. Between August 2018 and January 2019, individualised follow-up emails were sent to medical oncologists who had referred patients to the Molecular Screening and Therapeutics (MoST) Program [16]. The MoST Program is recruiting adult patients with advanced or metastatic solid cancers. Participants undergo CGP using an evolving range of panels including in-house assays (CCPv2, CCPv2.2, MoSTv1), the Illumina TruSight Tumor 170, and/or the Foundation Medicine panels, with the view to identify actionable findings that can inform novel treatment strategies.

### Data analysis

Descriptive statistics were used to summarise cohort characteristics and participants’ responses to survey items. For VAS, medians and IQR are reported given the variables were not normally distributed. Multiple linear regression was used to examine the association between VAS and participant baseline demographics (i.e., practice setting, gender and years of clinical experience). The Type I error rate was set at α = 0.01, accounting for family-wise error from multiple hypothesis testing and modelling. Chi-square testing was employed for survey questions with categorical responses. R environment version 3.5.3 was employed for statistical computing. Open-ended questions were analysed using content analysis [17].

## SUPPLEMENTARY MATERIALS




