# Supplementary material for: The experiences and needs of Australian medical oncologists in integrating comprehensive genomic profiling into clinical care: a nation-wide survey
Source: Oncotarget. 2021 Oct 12;12(21):2169–76. doi: 10.18632/oncotarget.28076 (PMC8522847; doi:10.18632/oncotarget.28076)
Supplement: Supplementary file 1 [file oncotarget-12-2169-s001.pdf]

The experiences and needs of Australian medical oncologists in integrating comprehensive genomic profiling into clinical care: a nation-wide survey

SUPPLEMENTARY MATERIALS

Supplementary Data: Clinician views on returning genomic results to cancer patients. See Supplementary Data

Supplementary Table 1: Multiple regression analysis showing association between clinical experience in referring patients for comprehensive genomic profiling (number of patients) and participating oncologists' demographics

Oncologist demographic factor	Estimate	95% CI	*P*
Years of experience as an oncologist (per 5 years)	0.0	(–0.1–0.2)	0.37
Urban practice	0.3	(–0.3–0.9)	0.30
Practicing in the state of New South Wales	0.3	(–0.2–0.8)	0.30
Male	0.1	(–0.4–0.6)	0.76

Adjusted R-squared: –0.01214, *p*-value: 0.5857.

Supplementary Table 2: Multiple regression analysis showing association between confidence to pursue an unproven therapy (100-point VAS response) and participating oncologists' demographics

Oncologist demographic factor	Estimate	95% CI	*P*
Years of experience as an oncologist (per 5 years)	4.8	(2.0–7.5)	**0.001**
Urban practice	8.2	(–5.6–22)	0.24
Practicing in the state of New South Wales	4.5	(–6.6–15.6)	0.42
Male	–10.1	(–21.1–0.9)	0.07

Adjusted R-squared: 0.08641, *p*-value (*F*-test): 0.00965.

Supplementary Table 3: Multiple regression analysis showing association between preference for clinician-based resources (100-point VAS response) and participating oncologists' demographics

Oncologist demographic factor	Estimate	95% CI	*P*
Years of experience as an oncologist (per 5 years)	–2.1	(–4.2– –0.1)	**0.04**
Urban practice	1.6	(–8.8–11.9)	0.77
Practicing in the state of New South Wales	1.6	(–6.5–9.8)	0.69
Male	–1.9	(–10–6.2)	0.64

Adjusted R-squared: 0.02515, *p*-value (*F*-test): 0.1595. Clinician-based resources include familial cancer centre helpline, genomics primer in text or video forms.
