# Supplementary material for: The experiences and needs of Australian medical oncologists in integrating comprehensive genomic profiling into clinical care: a nation-wide survey
Source: Oncotarget. 2021 Oct 12;12(21):2169–76. doi: 10.18632/oncotarget.28076 (PMC8522847; doi:10.18632/oncotarget.28076)
Supplement: Supplementary file 2 [file oncotarget-12-2169-s002.docx]

## **Supplementary Data: Clinician views on returning genomic results to cancer patients**

**Testing patients**

1. Have you ever sent patients for treatment-focused tumour genomic profiling or whole genome/exome sequencing?  Yes  No

If so, approximately how many?

 1-5  6-10  11-20  >20

1. Have you had patients undergo direct-to-consumer tumour testing independently and ask you to interpret their results?  Yes  No

If so, approximately how many?

 1-5  6-10  11-20  >20

1. Have you ever referred patients to a Family Cancer Clinic because of a strong family history?

 Yes  No

If so, approximately how many?

 1-5  6-10  11-20  >20

1. Have you had patients undergo direct-to-consumer germline testing independently (e.g. 23andMe) and ask you to interpret their results?  Yes  No

If so, approximately how many?

 1-5  6-10  11-20  >20

1. Do you think it is your role to interpret and discuss direct-to-consumer tumour testing results with patients?

 Yes  Yes, only if related to treatment  No  Not sure

Please give the reason(s) for your response: __________________________________________________________________________________________________________________________________________________________________________________________________________________________________________

If yes, what support, if any, would you need/like to assist with interpretation of results? __________________________________________________________________________________________________________________________________________________________________________________________________________________________________________

If not, whose role is it?  Geneticist  GP  Other (please specify) ___________________

1. Do you think there is a role for oncologists in ensuring clinical suitability for patients to undergo tumour genomic profiling?  Yes  No

If yes, please indicate which clinical parameters you would use:

 Life-expectancy ≥ 12 weeks

 Generally suitable for systemic therapy (no medical comorbidities or organ dysfunction that would preclude treatment)

 Reasonable performance status

 Other (please specify) ______________________________________________________

1. What would your advice be to a patient who requests treatment with an unproven targeted/ immunotherapy based on genetic testing (including high mutational burden/microsatellite instability (MSI)) before receiving standard therapies of proven efficacy?

__________________________________________________________________________________________________________________________________________________________________________________________________________________________________________

**Communicating results**

1. Do you feel it is your role to consent patients for tumour genomic profiling? Please indicate how strongly you agree or disagree on a scale from 0 to 10, with 0 being least agreeable.

| 0 | 1 | 2 | 3 | 4 | 5 | 6 | 7 | 8 | 9 | 10 |
| --- | --- | --- | --- | --- | --- | --- | --- | --- | --- | --- |
| Disagree | |  |  |  |  |  |  |  | Agree | |

Please explain your response: ____________________________________________________________________________________________________________________________________________________________

1. Do you feel it is your role to consent patients for germline genomic sequencing? Please indicate how strongly you agree or disagree on a scale from 0 to 10, with 0 being least agreeable.

| 0 | 1 | 2 | 3 | 4 | 5 | 6 | 7 | 8 | 9 | 10 |
| --- | --- | --- | --- | --- | --- | --- | --- | --- | --- | --- |
| Disagree | |  |  |  |  |  |  |  | Agree | |

Please explain your response: ____________________________________________________________________________________________________________________________________________________________

1. On a scale from 0 to 10, with 0 being least confident, how confident do you feel discussing the following with a patient:
2. process of tumour genomic profiling to obtain consent

| 0 | 1 | 2 | 3 | 4 | 5 | 6 | 7 | 8 | 9 | 10 |
| --- | --- | --- | --- | --- | --- | --- | --- | --- | --- | --- |
| Not at all  confident | |  |  |  |  |  |  |  | Extremely confident | |

1. probability of a “therapeutically actionable finding” based on tumour genomic profiling

| 0 | 1 | 2 | 3 | 4 | 5 | 6 | 7 | 8 | 9 | 10 |
| --- | --- | --- | --- | --- | --- | --- | --- | --- | --- | --- |
| Not at all  confident | |  |  |  |  |  |  |  | Extremely confident | |

1. tumour genomic profiling results

| 0 | 1 | 2 | 3 | 4 | 5 | 6 | 7 | 8 | 9 | 10 |
| --- | --- | --- | --- | --- | --- | --- | --- | --- | --- | --- |
| Not at all  confident | |  |  |  |  |  |  |  | Extremely confident | |

1. germline results

| 0 | 1 | 2 | 3 | 4 | 5 | 6 | 7 | 8 | 9 | 10 |
| --- | --- | --- | --- | --- | --- | --- | --- | --- | --- | --- |
| Not at all  confident | |  |  |  |  |  |  |  | Extremely confident | |

1. Do you think it is your role to discuss inherited cancer risk at the time of consent to tumour genomic profiling?  Yes  No

Please explain your response: __________________________________________________________________________________________________________________________________________________________________________________________________________________________________________

If yes, how confident do you feel discussing inherited cancer risk?

| 0 | 1 | 2 | 3 | 4 | 5 | 6 | 7 | 8 | 9 | 10 |
| --- | --- | --- | --- | --- | --- | --- | --- | --- | --- | --- |
| Not at all  confident | |  |  |  |  |  |  |  | Extremely confident | |

1. Do you think it is your role to discuss inherited cancer risk with family members when a germline finding is confirmed in an index case as a result of tumour genomic profiling?

 Yes, if present at the consultation

 Yes, regardless of whether present at the consultation

 No

Please explain your response: ____________________________________________________________________________________________________________________________________________________________

1. On a scale from 0 to 10, with 0 being least comfortable, how comfortable are you discussing inherited cancer risk with families?

| 0 | 1 | 2 | 3 | 4 | 5 | 6 | 7 | 8 | 9 | 10 |
| --- | --- | --- | --- | --- | --- | --- | --- | --- | --- | --- |
| Not at all comfortable | |  |  |  |  |  |  |  | Extremely comfortable | |

1. On a scale from 0 to 10, with 0 being least comfortable, how comfortable are you referring family members to a Family Cancer Clinic?

| 0 | 1 | 2 | 3 | 4 | 5 | 6 | 7 | 8 | 9 | 10 |
| --- | --- | --- | --- | --- | --- | --- | --- | --- | --- | --- |
| Not at all comfortable | |  |  |  |  |  |  |  | Extremely comfortable | |

**The report**

1. Have you previously received a tumour genomic profiling report?

 Yes  No

If yes, did it contain sufficient information for you to understand the results?  Yes  No

If No, what was missing? ______________________________________________________

If yes, was there more information provided than you required?  Yes  No

If Yes, what was superfluous? ___________________________________________________

1. What information do you think is essential to have on a tumour genomic profiling report?
   1. Please check one option only:

 All gene variants in cancer genes irrespective of clinical actionability

 Only those gene variants in cancer genes which are currently **clinically** actionable

 Only those gene variants in cancer genes which are currently **therapeutically** actionable

- 1. List of genes tested during tumour genomic profiling?  Yes  No
  2. Statement relating gene variants to recommended therapies?  Yes  No

If yes, what would you accept as a reliable information source? ______________________________________________________________________________________________________________________________________________________

- 1. List of available trials for Australian patients for the identified gene variant?

 Yes  No

If yes, what would you accept as a reliable information source? ______________________________________________________________________________________________________________________________________________________

- 1. Recommendation for referral to a Family Cancer Clinic for inherited gene variants (or likely inherited gene variants in the absence of tumour normal paired testing) in cancer predisposition genes?  Yes  No
  2. Would you like to have access to gene variants identified in genes not currently associated with cancer?

 Yes  Yes, but only if currently of clinical relevance  No

If yes:

What type of results would you want to have on the report? ____________________________________________________________________________________________________________________________________________

Would you prefer these separate to the main report e.g. as a link to additional information?  Yes, separate report  No, as part of same report

What type of advice would you require relating to these secondary findings? ____________________________________________________________________________________________________________________________________________

**Therapeutic options**

1. If you did not understand a component of the report, who would you consult?

 Oncology colleague

 Local Family Cancer Clinic staff

 Other (please specify) ________________________________________________

1. On a scale from 0 to 10, with 0 being least confident, how confident are you with using tumour genomic profiling results to obtain a targeted therapy for a patient – e.g. obtaining trastuzumab or T-DM1 for a patient with HER2-amplified endometrial cancer – outside of a trial setting?

| 0 | 1 | 2 | 3 | 4 | 5 | 6 | 7 | 8 | 9 | 10 |
| --- | --- | --- | --- | --- | --- | --- | --- | --- | --- | --- |
| Not at all  confident | |  |  |  |  |  |  |  | Extremely confident | |

- 1. What are the factors that are involved in making this decision?

__________________________________________________________________________

- 1. Are you more likely to pursue this in a treatment-refractory patient?  Yes  No
  2. Is there a minimal level of evidence (even in the treatment-refractory setting) you would require before pursuing this therapy outside of the trial setting?  Yes  No
  3. What level of evidence would you require?

 Effective in another cancer type is sufficient

 Marker of effectiveness seen in ‘solid tumours’ is sufficient

 Require evidence in the relevant tumour type, with either prospective or retrospective selection based on this particular molecular characteristic

 Other (please specify) _____________________________________________________

**Resources**

1. If available, would you use any of these resources to help with communication of genomic test results? Please indicate how likely you would be to use the stated resource on a scale from 0 to 10, with 0 being least likely.
2. Family Cancer Clinic helpline for patients

| 0 | 1 | 2 | 3 | 4 | 5 | 6 | 7 | 8 | 9 | 10 |
| --- | --- | --- | --- | --- | --- | --- | --- | --- | --- | --- |
| Not at all likely | |  |  |  |  |  |  |  | Extremely likely | |

1. Family Cancer Clinic helpline for clinicians

| 0 | 1 | 2 | 3 | 4 | 5 | 6 | 7 | 8 | 9 | 10 |
| --- | --- | --- | --- | --- | --- | --- | --- | --- | --- | --- |
| Not at all likely | |  |  |  |  |  |  |  | Extremely likely | |

1. Online primer on genomics (text)

| 0 | 1 | 2 | 3 | 4 | 5 | 6 | 7 | 8 | 9 | 10 |
| --- | --- | --- | --- | --- | --- | --- | --- | --- | --- | --- |
| Not at all likely | |  |  |  |  |  |  |  | Extremely likely | |

1. Online primer on genomics (video)

| 0 | 1 | 2 | 3 | 4 | 5 | 6 | 7 | 8 | 9 | 10 |
| --- | --- | --- | --- | --- | --- | --- | --- | --- | --- | --- |
| Not at all likely | |  |  |  |  |  |  |  | Extremely likely | |

1. Downloadable patient information sheets on genomic testing and its implications

| 0 | 1 | 2 | 3 | 4 | 5 | 6 | 7 | 8 | 9 | 10 |
| --- | --- | --- | --- | --- | --- | --- | --- | --- | --- | --- |
| Not at all likely | |  |  |  |  |  |  |  | Extremely likely | |

1. Downloadable patient decision aids

| 0 | 1 | 2 | 3 | 4 | 5 | 6 | 7 | 8 | 9 | 10 |
| --- | --- | --- | --- | --- | --- | --- | --- | --- | --- | --- |
| Not at all likely | |  |  |  |  |  |  |  | Extremely likely | |

1. Are there other resources you would use? ___________________________________________________________________________

**Demographics**

1. How many years have you worked as an oncologist?

 0-4  5-9  10-14  15-19  20-24  25-29  30-34  35-39  ≥ 40

1. Is your primary practice urban or regional?

 Urban  Regional

1. Which state do you work in?

 Australian Capital Territory  New South Wales  Northern Territory

 Queensland  South Australia  Tasmania  Victoria  Western Australia

1. Sex  M  F
2. Age (years)

 20-29  30-39  40-49  50-59  60-69  ≥70
